# Insulin-like growth factor 2 is a key mitogen driving liver repopulation in mice

**DOI:** 10.1038/s41419-017-0186-1

**Published:** 2018-01-18

**Authors:** Min-Jun Wang, Fei Chen, Qing-Gui Liu, Chang-Cheng Liu, Hao Yao, Bing Yu, Hai-Bin Zhang, He-Xin Yan, Yibiao Ye, Tao Chen, Kirk J. Wangensteen, Xin Wang, Yi-Ping Hu, Zhi-Ying He

**Affiliations:** 1 Department of Cell Biology, Center for Stem Cell and Medicine, Second Military Medical University, Shanghai, 200433 China; 2 Translational Medical Center for Stem Cell Therapy & Institute for Regenerative Medicine, Shanghai East Hospital, School of Life Sciences and Technology, Tongji University, Shanghai, 200123 China; 3 Eastern Hepatobiliary Surgery Hospital, Second Military Medical University, Shanghai, 200433 China; 4 Department of Anesthesia and Intensive Medicine, Renji Hospital Affiliated to Shanghai Jiaotong University School of Medicine, Shanghai, 200127 China; 5 Department of Hepatobiliary Surgery, Sun Yat-sen Memorial Hospital, Sun Yat-sen University, Guangzhou, China; 6 Department of Medicine, Division of Gastroenterology, University of Pennsylvania, Philadelphia, PA 19104 USA; 7 The Key Laboratory of National Education Ministry for Mammalian Reproductive Biology and Biotechnology, Inner Mongolia University, Huhhot, 010070 China; 8 Department of Laboratory Medicine and Pathology, University of Minnesota, Minneapolis, MN 55455 USA; 9 Hepatoscience Incorporation, 725 San Aleso Avenue, Sunnyvale, CA 94085 USA

## Abstract

Hepatocyte transplantation holds great promise as an alternative to orthotopic organ transplantation in the treatment of liver diseases. However, obtaining clinically meaningful levels of liver repopulation has not been achieved because the mechanisms regulating hepatocyte proliferation in recipient livers have not yet been well characterized. In the mouse model of Hereditary Tyrosinemia Type I, the fumarylacetoacetate hydrolase-deficient (*Fah*^−/−^) mouse, we found gradually increasing expression level of insulin-like growth factor 2 (IGF2) in the hepatocytes of host livers. Similarly, high levels of IGF2 were found in the livers of patients with deficient FAH activity. Recombinant IGF2 directly promotes proliferation of primary hepatocytes in vitro. Inhibition on IGF2 expression through the interruption of PI3K/Akt and MAPK pathways significantly reduced the level of liver repopulation in *Fah*^−/−^ mice. Interestingly, treatment with IGF2 before hepatocyte transplantation generally improved the amount of liver repopulation seen in various mice models of liver injury. Altogether, these findings underscore the underlying mechanisms of therapeutic liver repopulation in *Fah*^−/−^ mice, and indicate that IGF2 is a potential hepatocyte mitogen for liver cell transplantation therapies.

## Introduction

Cell transplantation therapies have the potential to treat a wide variety of diseases by making up for tissue defects. Several obstacles still hinder the widespread clinical application of cell therapies. Most importantly, the difficulty in achieving sufficient donor cell engraftment into host tissues is one major technical obstacle^1^. Hepatocyte transplantation therapy has been performed in clinical trials as an alternative to orthotopic liver transplantation for some types of genetic diseases of the liver and for acute liver failure^2,3^. However, the extent of liver engraftment and repopulation after hepatocyte transplantation was very limited. Therefore, technological improvements to improve therapeutic liver repopulation could lead to successes in cell therapy for liver diseases.

Indeed, therapeutic liver repopulation can be examined under experimental conditions in animal models^4–9^. Two strategies have been successfully applied. The first is to suppress the proliferative capacity of host hepatocytes through inducing cell injuries or damages^4–7^. The second is to supply or regulate hepatic mitogens as well as cell-cycle regulators to drive proliferation of the transplanted hepatocytes in recipient livers^8,9^. Among the rodent models for liver repopulation, the mouse model of Hereditary Tyrosinemia Type I (HT1), the fumarylacetoacetate hydrolase-deficient (*Fah*^−/−^) mouse, is the best example of repopulation of the liver, reaching >90% of total hepatocyte replacement by transplanted wild-type hepatocytes^10,11^.

The liver failure observed in *Fah*^−/−^ mouse is similar to what is seen in humans with HT1^10^. Loss of FAH results in famarylacetoacetate (FAA) accumulation, a major toxic metabolite, which causes extensive and continuous hepatocyte injury. 2-(2-nitro-4-trifluoro-methyl-benzoyl)-1, 3-cyclohexanedione (NTBC) inhibits accumulation of toxic metabolites in hepatocytes to maintain *Fah*^−/−^ mice in a healthy state. However, the underlying molecular mechanisms and factors responsible for high repopulation in *Fah*^−/−^ mice still remain elusive and are not well defined. Results from previous studies found that hepatocytes in the livers of *Fah*^−/−^ mice undergo DNA damage^12^. Furthermore, a genetic screen has been performed to reveal Foxa3 and TNFR1 as a strong promoter and suppressor of liver repopulation in *Fah*^−/−^ mice^13^. However, it is not known whether some mitogens are expressed by injured host hepatocytes to enhance the proliferative capacity of transplanted hepatocytes in *Fah*^−/−^ mice.

The objective of this study is to carefully elucidate the mechanism of therapeutic liver repopulation in *Fah*^−/−^ mouse, which could be used to achieve therapeutic liver repopulation in clinical settings. In the present study, we analyzed the pathological changes in the liver tissues of *Fah*^−/−^ mice undergoing injury due to tyrosinemia to discover potential hepatic mitogens which could promote hepatocyte proliferation. We found that the hepatocytes undergoing injury gradually upregulate IGF2 to high levels. Interestingly, IGF2 expression levels return to normal when liver repopulation is completed. Provision of exogenous IGF2 proved it to be an effective mitogen for promotion of proliferation of transplanted hepatocytes. Conversely, inhibition of IGF2 production inhibited repopulation. These findings indicate that IGF2 therapy is a potential strategy promoting liver repopulation in clinical settings.

## Results

### IGF2 expression is induced during liver injury in *Fah*^−/−^ mice

**Fig. 1:**
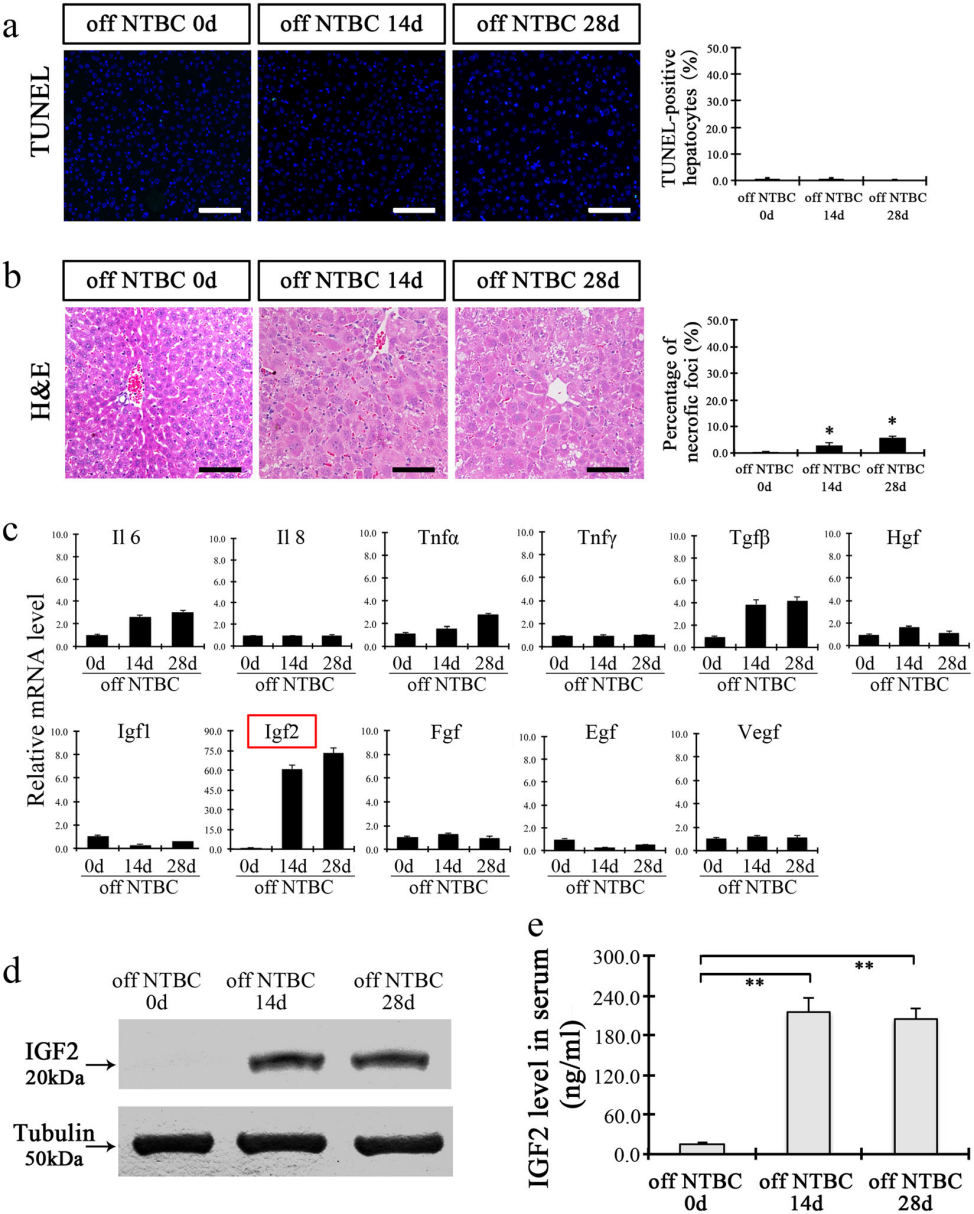
IGF2 is upregulated during liver injury in *Fah*^−/−^ mice. Individual panels show the representative photographs for TUNEL staining (**a**), and hematoxylin-eosin (H&E) staining (**b**) in the livers of *Fah*^−/−^ mice withdrawal of NTBC for 0, 14, and 28 days. The right bar graphs show the quantification of TUNEL-, and necrotic foci-positive cells. **c** Quantitative RT-PCR of growth factors and cytokines at 0, 14, and 28 days’ livers after NTBC withdrawal. **d** IGF2 expression levels in liver tissues of *Fah*^−/−^ mice at 0, 14, and 28 days after NTBC withdrawal. (**e**) Quantitation of circulating IGF2 levels in *Fah*^−/−^ mice at 0, 14, and 28 days after NTBC withdrawal. **p < 0.01. Shows are mean ± S.D. Scale bar, 100 µm

The hepatocytes of *Fah*^−/−^ mice undergo injury upon termination of NTBC administration. However, in line with previous reports^14^, we found that only a few scattered hepatocytes become positive for the assay of terminal deoxynucleotidyl transferase-mediated deoxyuridine triphosphate nicked labeling (TUNEL), and only a few small necrotic foci were found in the livers of *Fah*^−/−^ mice off NTBC for up to 4 weeks (Fig. 1a, b). These results indicated that there is a lack of cell death of host hepatocytes at the initial stages after hepatocyte transplantation in *Fah*^−/−^ mice, implying that hepatic mitogens released by these cells might be responsible for successful liver repopulation in *Fah*^−/−^ mice.

Previously, several growth factors and cytokines, such as IL6, TNFα, FGF, and HGF, were found to act as mitogens to promote hepatocyte proliferation following partial hepatectomy^15–17^. To test the hypothesis that specific mitogens may promote hepatocyte repopulation of *Fah*^−/−^ mice, the candidate factors were investigated in liver tissue by performing RT-PCR at various time points after removal of NTBC (Fig. 1c). Interestingly, Igf2 expression was found to increase 60-fold at 14 days after withdrawal of NTBC, and increased even more at 28 days. There was a corresponding increase in level of IGF2 protein (Fig. 1d). Remarkably, the level of IGF2 protein in the serum was also found to increase after withdrawal of NTBC (Fig. 1e).

### IGF2 is expressed in the hepatocytes of *Fah*^−/−^ mice and HT1 patients

**Fig. 2:**
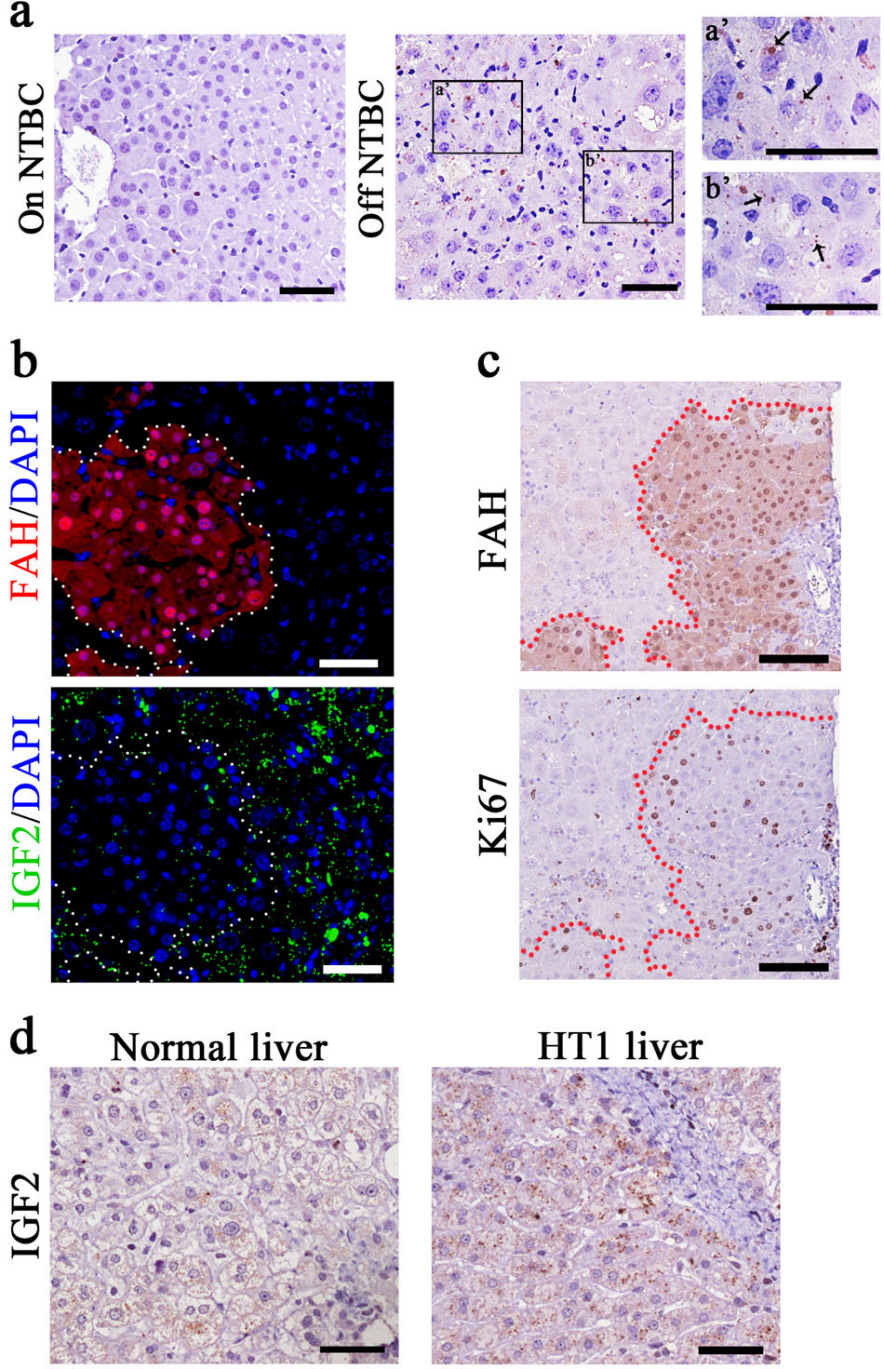
IGF2 is expressed in host hepatocytes of *Fah*^−/−^ mice and in a human HT1 patient. **a** IGF2 protein (arrows) was found in liver sections of NTBC withdrawal mice (a′, b′: the magnification). Mice on NTBC were used as control. Scale bar, 50 µm. **b** Expression of IGF2 (green) in repopulated livers (red) with transplanted hepatocytes. The region inside dashed line is repopulated nodules from transplanted hepatocytes (FAH^+^), and the region outside dashed line is the host hepatocytes in the liver of *Fah*^−/−^ mice (FAH^−^). Scale bar, 50 µm. **c** Immunohistochemistry assays analyzed on the parameters of Ki67 and FAH in repopulated livers at 4 weeks after hepatocyte transplantation. Serial sections were used for Ki67 and FAH staining in repopulated livers. Scale bar, 100 µm. (**d**) Immunohistochemistry assay of IGF2 in liver of normal controls (30 years) and an 8-year-old patient with HT1. **p < 0.01. Shows are mean ± S.D. Scale bar, 50 µm

We examined which cell type in the liver is the major source of IGF2 expression after liver injury. The results indicated that IGF2 was localized to the cytoplasm hepatocytes, and was not detected in other cell types. Furthermore, IGF2 protein was present in the hepatocytes of *Fah*^−/−^ mice only after withdrawal of NTBC (Fig. 2a). Moreover, immunohistochemistry assays of repopulated livers demonstrated that IGF2 expression surrounded repopulation nodules in the host liver cells, whereas no expression was detected in FAH enzyme-expressing repopulation nodules (Fig. 2b). Next, DNA synthesis in these FAH^+^ hepatocytes was analyzed through the detection of Ki67 protein on the serially sectioned liver samples. Results revealed that numerous hepatocytes were positive for both Ki67 and FAH inside repopulated nodules, whereas very few of the surrounding host FAH^−^hepatocytes were positive for Ki67 (Fig. 2c). These data confirm that IGF2 is induced in the injured FAH^−^deficient host hepatocytes and suggesting that it may act in paracrine fashion on the repopulating, wild-type donor hepatocytes.

Next, we investigated whether IGF2 expression was induced in the humans with liver injury. Whereas few hepatocytes were positive for IGF2 expression in a healthy control, the expression of IGF2 was significantly induced in the hepatocytes of an 8-year-old patient with HT1 (Fig. 2d). These data suggest that increased expression of IGF2 in liver injury may promote liver repopulation.

### IGF2 promoted the proliferation of both primary hepatocytes in vitro and transplanted hepatocytes in vivo

**Fig. 3:**
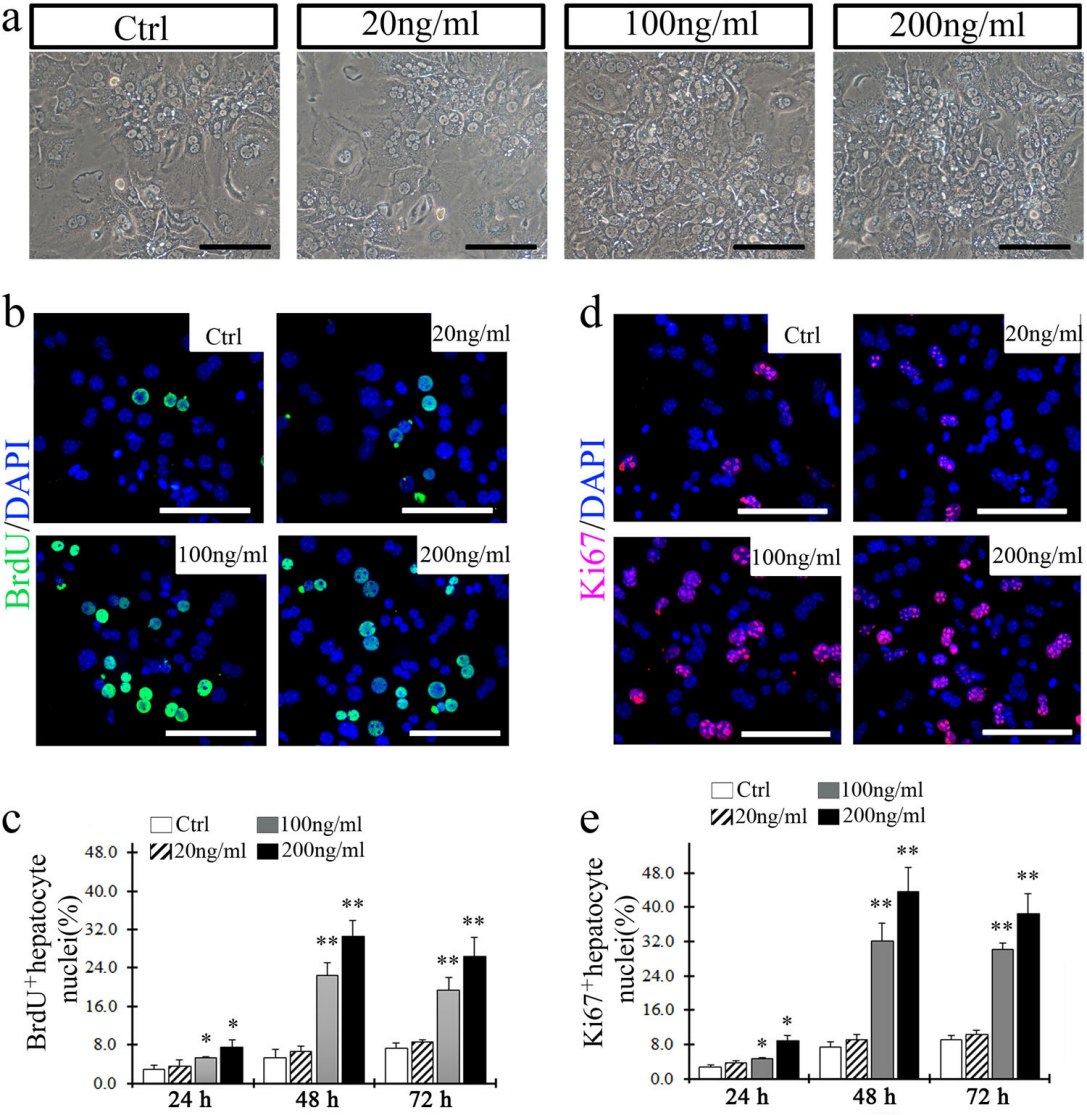
IGF2 promotes the proliferation of wild-type hepatocytes in culture. **a** Microscopic images showed the density and morphology for the cultured hepatocytes with or without IGF2 stimuli at 48 h. **b** Primary hepatocytes were BrdU-positive after treatment with IGF2 for 48 h. **c** Quantification of BrdU-positive hepatocytes cultured by 24, 48, and 72 h after stimulation with IGF2 (20, 100, 200 ng/ml). **d** Ki67-positive primary hepatocytes were found at 48 h after IGF2 treatment. **e** Quantification of Ki67-positive hepatocytes. *p *<* 0.05, **p *<* 0.01 vs. cultured hepatocytes without IGF2 stimuli. Shows are mean ± S.D. Scale bar, 100 µm

IGF2 was previously reported to act as a hepatocyte mitogen by promoting DNA synthesis^18^. To examine whether IGF2 could affect hepatocyte proliferation directly, we performed a growth assay using wild-type primary hepatocytes cultured with increasing amounts of IGF2 added to the media. The results indicated that 100 and 200 ng/ml of IGF2 stimulated hepatocyte proliferation significantly (Fig. 3a). Interestingly, this concentration matches the level of IGF2 found in the serum of *Fah*^−/−^ mice after NTBC withdrawal. We confirmed stimulated proliferation by performing a BrdU assay, which confirmed that BrdU-positive hepatocytes were significantly increased with IGF2 treatment for 48 h (Fig. 3b, c). A similar result was found using Ki67 immuno-staining (Fig. 3d, e).

**Fig. 4:**
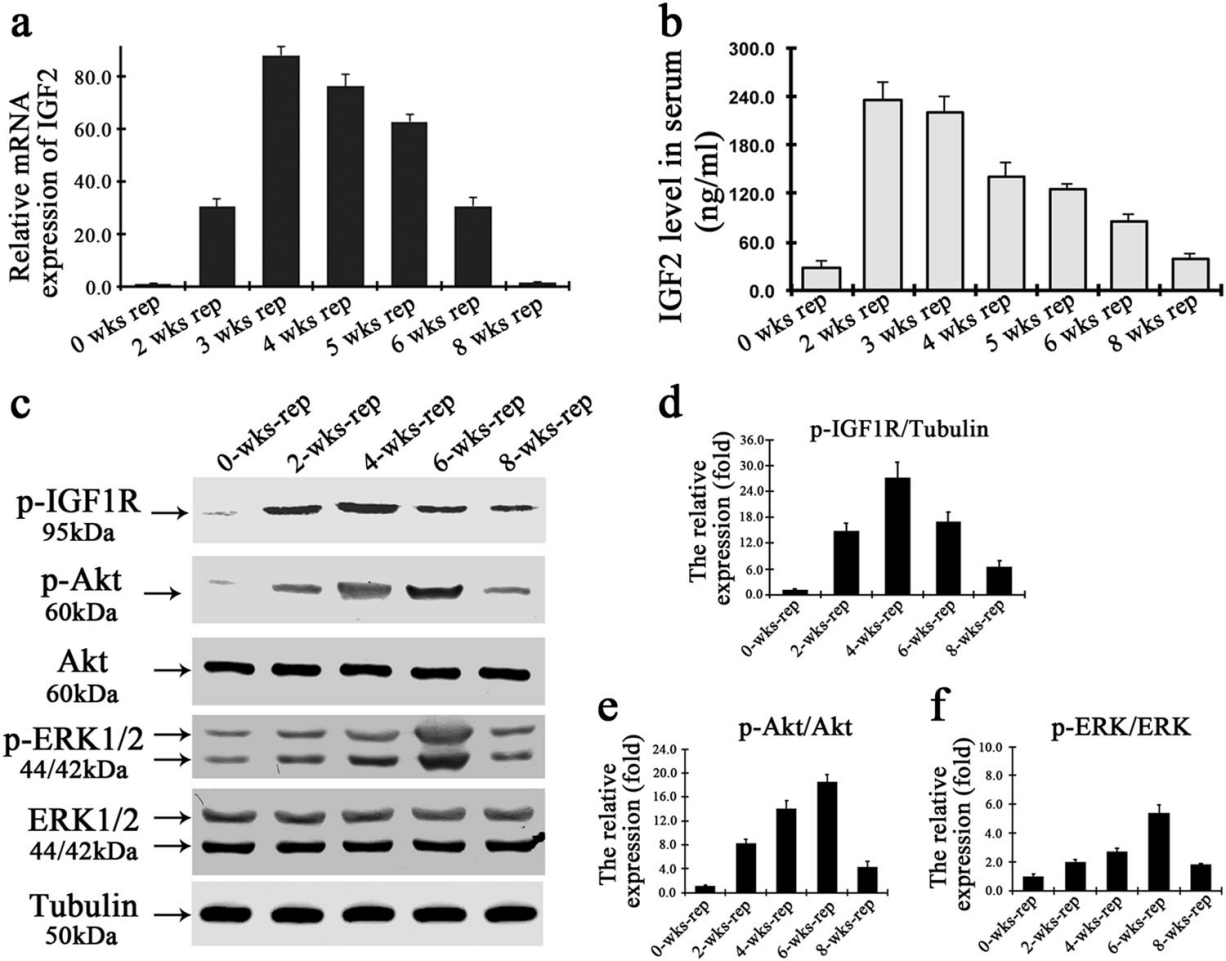
IGF2 promotes the proliferation of transplanted hepatocytes through activation of PI3K and MAPK pathways. **a**, **b** Quantitative assays on the Igf2 mRNA (**a**) and circulating IGF2 protein (**b**) in *Fah*^−/−^ mice after hepatocyte transplantation. **c** Western blot analysis of phosphorylated IGF1R, extracellular signal-regulated kinase (ERK), and AKT in repopulated livers after hepatocytes transplantation. **d** The graph data is the relative level of phosphorylated IGF1R normalized to the inner reference tubulin. **e**, **f** Graphs summarizing relative protein expression levels of phosphorylated AKT (**e**) or ERK (**f**) normalized to AKT or ERK, respectively, presented in relative ratios and mean ± S.D., **p *<* 0.01

IGF2 is known to bind to insulin-like growth factor receptor 1 (IGF1R), which activates downstream members of PI3K/AKT and mitogen-activated protein kinase (MAPK) pathways^19-21^. To investigate the potential role of IGF2 on liver repopulation, the IGF2 level was analyzed in the repopulated liver of *Fah*^−/−^ mice after hepatocyte transplantation. Both mRNA and protein levels of IGF2 increased significantly at the initial stage of liver repopulation within 3 weeks after hepatocyte transplantation (Fig. 4a, b). The expression level then gradually decreased after 3 weeks, and returned to the basal level upon completion of liver repopulation (Fig. 4a, b). Remarkably, there was increased of the phosphorylation of IGF1R and its effectors such as ERK1/2 and AKT in the proliferating hepatocytes, and the levels gradually decreased as liver repopulation went to completion (Fig. 4c–f).

These data indicate that IGF2 is a paracrine signal from the injured hepatocytes that enhances the proliferative capacity of repopulating donor hepatocytes through the activation of AKT and ERK1/2 signaling pathways.

### Inhibition of IGF2 expression during hepatocyte transplantation decreased liver repopulation

To examine whether IGF2 expression is required to promote liver repopulation in livers of *Fah*^−/−^ mice, we targeted the Igf2 gene using adenovirus vectors expressing shRNA targeting Igf2 (Ad-shIGF2). We used an adenovirus vector expressing GFP gene as a control (Ad-shRNA control). We designed four Ad-shIGF2 vectors, and first tested them in vitro. The Ad-shIGF2 vector with the highest knockdown efficiency was selected for in vivo studies (Supplementary Figure 1).

**Fig. 5:**
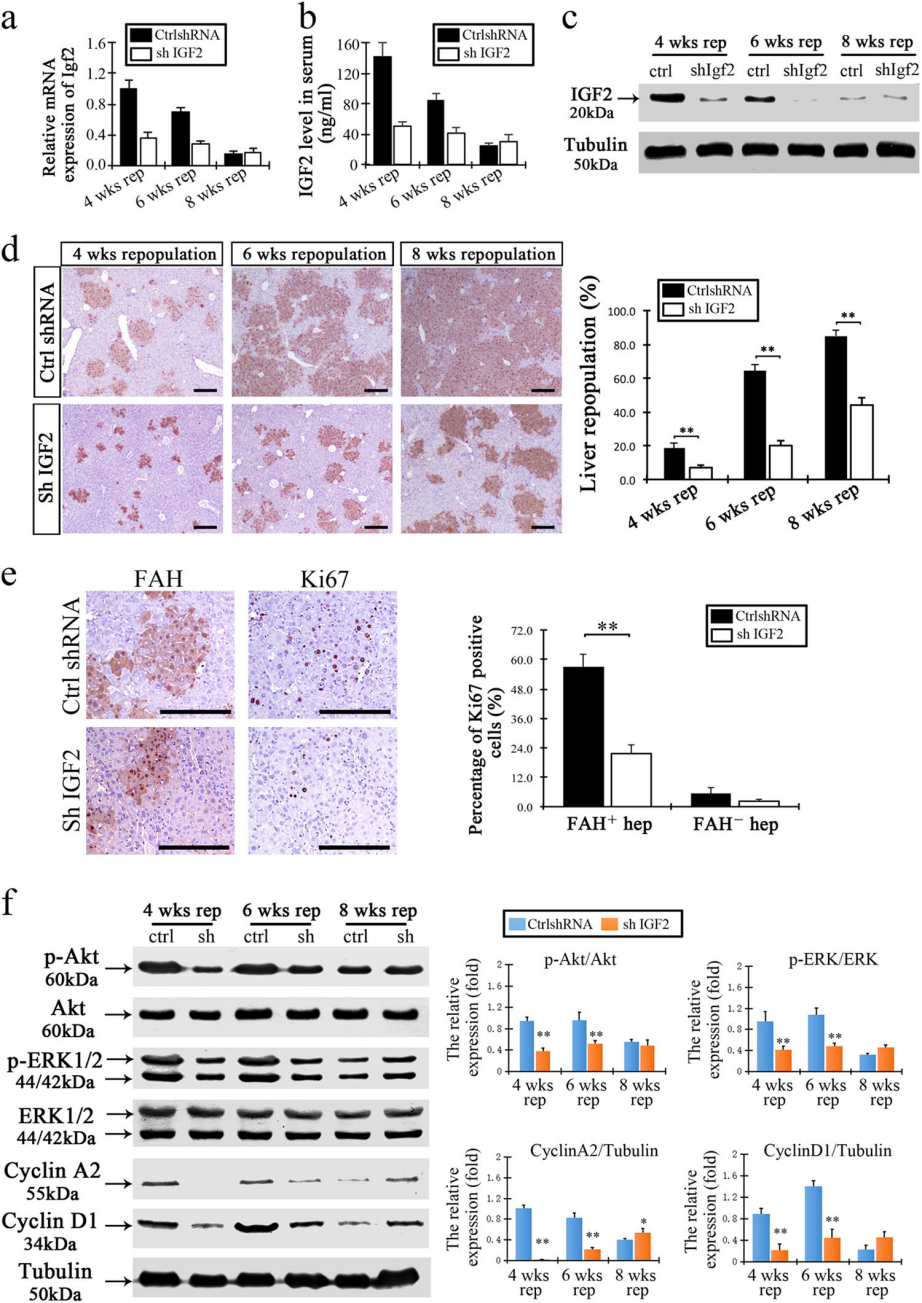
Inhibition of IGF2 expression decreased the levels of liver repopulation. **a**, **b** Levels of IGF2 for both mRNA expression (**a**) and circulating concentration (**b**) in the livers of Ad-shIGF2-treated mice decreased after hepatocytes transplantation. **c** Levels of IGF2 protein expression decreased in the repopulating livers of Ad-shIGF2-treated mice at 2 at 4, 6, and 8 weeks after hepatocyte transplantation. **d** Representative sections showed FAH-positive hepatocytes in the repopulated livers at 4, 6, and 8 weeks after hepatocyte transplantation. The right panel showes the proportion of liver repopulation in the Ad-control-shRNA or Ad-shIGF2-treated mice. **e** The numbers of FAH-positive and Ki67-positive hepatocytes decreased in the livers of mice with 4 weeks of Ad-shIGF2 treatment. The right graph showed the percentage of Ki67-positive cells in both FAH-positive repopulated hepatocytes and FAH negative host hepatocytes after Ad-shIGF2 treatment. **f** Levels of p-AKT, AKT, p-ERK1/2, ERK1/2, cyclin A2, and cyclin D1, respectively, in the livers of mice with Ad-shIGF2 treatment at 4, 6, and 8 weeks. The right graphs showed the relative levels of protein expression. Phosphorylated AKT and ERK1/2 were normalized to AKT and ERK1/2 respectively. Cyclin A2 and cyclin D1 normalized to tubulin. Values of Ad-control-shRNA treatment was set as the baseline and considered equal to 1. Data are presented as relative ratios and mean ± S.D. *p *<* 0.05, **p *<* 0.01 vs. Ad-control-shRNA-treated livers. Scale bar, 200 µm

We injected *Fah*^−/−^ mice with Ad-shIGF2 or control after transplantation of donor hepatocytes. Liver samples were harvested at 4, 6, and 8 weeks of repopulation. The levels of both intracellular IGF2 (mRNA and protein) and circulating IGF2 (protein) were significantly decreased in the Ad-shIGF2-treated mice (Fig. 5a–c). Interestingly, the level of liver repopulation observed in the Ad-shIGF2-treated mice was substantially reduced at each time point when compared to controls (7.23 ± 1.44, 19.8 ± 3.32, and 44.2 ± 4.29 vs. 18.57 ± 2.81%, 64.23 ± 3.72%, and 85.03 ± 3.29%, respectively, Fig. 5d). We next examined proliferation in the liver tissue and found that the number of Ki67-positive hepatocytes was significantly decreased in the repopulation nodules when IGF2 was knocked down (Fig. 5e). In addition, we investigated the activation level of AKT and ERK1/2 in the two treatment groups. The results indicated that there was a decrease in phosphorylation levels for both AKT and ERK1/2 with IGF2 knockdown, which suggested IGF2 signaling is required for activations of these pathways in repopulating hepatocytes (Fig. 5f).

Collectively, above results indicate that IGF2 enhances liver repopulation with transplanted hepatocytes in *Fah*^−/−^ mice.

### Promotion of liver repopulation with IGF2 in various animal models

To study whether supplementation of IGF2 could enhance the proliferative capacity of transplanted hepatocytes and improve liver repopulation, we determined the effect of IGF2 treatment on other models of liver injury, including retrorsine (RS) plus 2/3 partial hepatectomy (PH) or CCl_4_ treatment. First, the induced mRNA expression for Igf2 along with some other growth factors were analyzed in the livers treated with RS plus 2/3 PH or CCl_4_. We found no significantly increased levels of these candidate growth factors including IGF2, indicating that high IGF2 expression may be unique to certain situations such as in HT1 (Supplementary Figure 2).

**Fig. 6:**
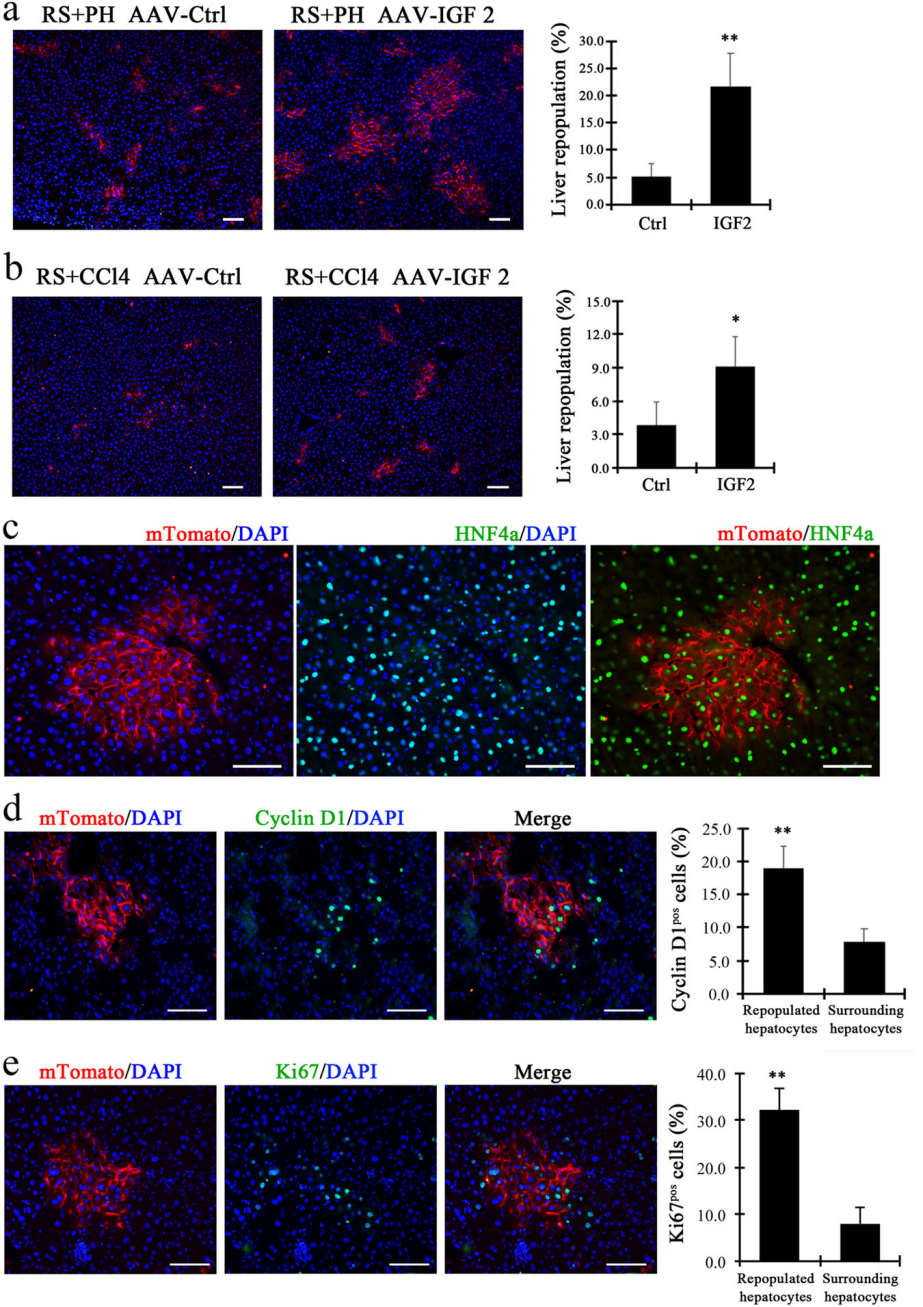
IGF2 promotes liver repopulation in other mouse models of liver injury. **a**, **b** The amounts of ROSA^mT/mG^ donor hepatocytes (mTomato-positive) increased in the livers of recipient mice treated with RS followed by 2/3 PH (**a**) or those treated with RS followed by CCl_4_ treatment (**b**). The right panel showed the percentages of mTomato^+^ hepatocytes in the livers of mice with or without AAV IGF2 gene delivery. **c** Co-localization through co-staining with mTomato and HNF4a antibodies indicated the repopulated nodules. **d**, **e** Co-localization through co-staining with cyclin D1 (**d**) or Ki67 (**e**) and mTomato in repopulated livers after hepatocyte transplantation. *p < 0.05, **p *<* 0.01. Shows are mean ± S.D. Scale bar, 100 µm

Next, IGF2 was overexpressed in the hepatocytes of RS-treated mice using an adeno-associated virus (AAV)-mediated gene delivery system. ROSA^mT/mG^ donor hepatocytes, which were labeled with membrane-localized tdTomato (mT) fluorescence, were transplanted into two groups of mice recipients: (1) mice treated with RS followed by 2/3 PH; (2) the mice treated with RS followed by CCl_4_ injection. Four weeks after transplantation, liver samples were harvested and examined for liver repopulation. The results indicated that AAV-mediated IGF2 gene delivery increased the level of liver repopulation by 21.75 ± 5.91% in mice treated with RS followed by 2/3 PH (Fig. 6a). This occurred via an increase in size of repopulation nodules (Fig. 6a). Similarly, ectopic IGF2 expression increased the level of liver repopulation and the size of repopulated nodules in mice treated with RS followed by CCl_4_ injection (Fig. 6b). The mTomato-positive donor hepatocytes expressed HNF4α, a hepatocyte marker, during liver repopulation, indicating they are bona fide hepatocytes (Fig. 6c). In addition, numerous of the mTomato-positive hepatocytes were positive for cyclin D1, whereas fewer surrounding host hepatocytes were positive for cyclin D1 (Fig. 6d). Similar results were seen with Ki67 (Fig. 6e).

In summary, overexpression of IGF2 promoted liver repopulation in liver injury models other than *Fah*^−/−^ mice. The results also imply that IGF2 could be used to improve liver repopulation in clinical application of hepatocyte transplantation.

## Discussion

Although hepatocyte transplantation is currently considered an experimental alternative to orthotopic liver transplantation in a few special circumstances, therapeutic levels of liver engraftment repopulation have been hard to reach in most clinical cases. Theoretically, successful liver repopulation will depend on an optimal combination of events, including sufficient blockage of host hepatocyte proliferation in the recipient liver, combined with stimulated proliferation of the transplanted hepatocytes to enable them to win a competition with host hepatocytes for repopulation of the liver^5,22^. In some patients with acute liver failure, rapid proliferation of transplanted hepatocytes in a short time is the key step for replenishing the missing metabolic capacity. Although repeated transplantation of hepatocytes in large numbers could increase the engraftment of transplanted cells and enhance the liver repopulation, this approach has a risk of thrombosis. In addition, transplanted human hepatocytes must be isolated from unused or rejected donor organs and either used immediately or cryopreserved, therefore there is a shortage of hepatocytes of good quality and enough quantity for transplantation. If the transplanted hepatocytes could be stimulated by mitogenic factors to reach massive levels of repopulation within a short time, the utility in acute liver failure would be tremendous.

Multiple signaling pathways are known to become activated after PH, when including several mitogenic growth factors^23^. Among these, HGF is regarded as a “complete mitogen”, as it can induce DNA synthesis during liver regeneration. VEGF promotes hepatocyte proliferation through stimulating HGF production. Some circulating growth factors including EGF and TGFα, produced by hepatocytes, stimulate liver regeneration in autocrine or paracrine fashion. TNF as a pro-inflammatory cytokine mediates hepatocyte apoptosis during PH induced liver regeneration. However, the mitogenic growth factors identified in PH may not apply to other types of liver “regeneration”, such as the liver repopulation that occurs in *Fah*^−/−^ mice. In the present study, we examine mitogens in *Fah*^−/−^ undergoing liver injury and repopulation, by examining changes in mRNA levels in *Fah*^−/−^ mouse livers. Among these, IGF2 was to have high levels of induction during liver injury. Interestingly, the expression level of IGF2 increases just slightly during liver regeneration after PH^18,19^, reflecting differences between PH and liver injury in *Fah*^−/−^ mice. Our study systematically analyzes IGF2 activation during liver injury in *Fah*^−/−^ mice, and indicated that IGF2 is a hepatocyte mitogen which promotes liver repopulation. However, other as yet uncharacterized mitogens could also contribute to the promotion on liver repopulation, as shRNA blockade of IGF2 did not fully block the hepatocyte repopulation in *Fah*^−/−^ mice. It will be necessary to characterize these as yet unknown hepatocyte mitogens in future studies.

The results from a recent report indicated that short-term treatment with growth hormone improves liver repopulation, which augmented the therapeutic benefit of clinical hepatocyte transplantation^24^. The findings also suggested the possible clinical application of IGF2 during hepatocyte transplantation, which was supported by our findings that overexpression of IGF2 improved liver repopulation in several models of liver injury. In both mouse and human, IGF2 is highly expressed in fetal liver, but the expression level is markedly downregulated in the quiescent adult liver^25,26^ Interesting, it is reported that supplementation of IGF2 into the hippocampus of old rats or mice enhances memory and rescues behavioral deficits^27,28^. These data suggest that supplementation of IGF2 could be safe and practical in clinical hepatocyte transplantation.

In summary, host hepatocytes of *Fah*^−/−^ mice undergoing injury upregulate IGF2, which stimulates repopulation of the liver by donor hepatocytes. Importantly, IGF2 activates PI3K/AKT and MAPK pathways in transplanted hepatocytes. Our study identifies a key mechanism driving therapeutic liver repopulation in *Fah*^−/−^ mice, and suggests a practical strategy to efficiently expand hepatocytes transplanted into receipients in clinical settings.

## Materials and methods

### Animals

*Fah*^−/−^ mice (6–8-weeks old) and wild-type donor mice were on a 129S4 background. ROSA-mTomato/mGFP (ROSA^mT/mG^) reporter mice and wild-type recipients were maintained on a C57BL/6 background. *Fah*^−/−^ mice were maintained with or without 7.5 mg/l 2-(2-nitro-4-trifluoromethylbenzoyl)-1, 3-cyclohexanedione (NTBC) in the drinking water^10,11^. Human liver tissue was obtained from a surgical resection specimen of a patient with a hepatic hemangioma and a patient with HT1, and consent from the patients were obtained. All procedures and protocols were according to institutional guidelines.

### Hepatocyte isolation, cell transplantation, and repopulation assay

Mouse livers were perfused with collagenase D (Roche, Indianapolis, IN) solution as previously described^29,30^. Isolated hepatocytes were injected intrasplenically into recipient mice as described previously^29^. After hepatocytes transplantation, livers were harvested and immunohistochemistry with FAH antibody or mTomato was used to examine the percentage of liver repopulation as described previously^29^.

### Experimental liver treatments

For retrosine-based 2/3 PH injury model^31^, C57BL/6 wild-type mice were given three injections of retrorsine (Sigma-Aldrich, St. Louis, MO), 30 mg/kg each, intraperitoneally, 3, 2, and 1 weeks before the transplantation. 1 day before the transplantation, recipient mice were subjected to a two-thirds partially hepatectomy (PH). For retrosine-based CCl_4_ injury^32^, C57BL/6 recipient mice were intraperitoneally infused with 0.6ul/g carbon tetrachloride (CCl_4_, Sigma-Aldrich) 1 day before the transplantation.

### Primary hepatocyte culture and BrdU incorporation

Isolated primary hepatocytes were plated on collagen-coated 6-well plates in Hepatic Cell Culture Medium (Invitrogen, Carlsbad, CA). Six hours after seeding, non-adherent cells were removed by washing the plated with phosphate buffered saline and the attached cells were then cultured in conditional hepatic cell culture medium supplemented with 0, 20, 100, and 200 ng/ml recombinant IGF2 (R&D Systems, Minneapolis, MN). For BrdU incorporation assay, cells were incubated for 24 h with BrdU regent (final concentration 100 μM) (Sigma-Aldrich). Then, cells were fixed with a 1:1 mixture of ice-cold methanol:glacial acetic acid after washing with PBS, and then incubated with 2 N HCl for 30 min at room temperature. Then cells were incubated with primary and second antibody as with the IHC protocols.

### Construction adenoviruses expressing Igf2 shRNA

Small hairpin RNA (shRNA) sequences for the Igf2 gene were chosen from Sigma-Aldrich and listed as follows: Igf2 shRNA-1:5′-CCGGGCTTGTTGACACGCTTCAGTTCTCGAGAACTGAAGC

GTGTCAACAAGCTTTTTG-3′, 5′-AATTCAAAAAGCTTGTTGACACGCTTCAGTTCT

CGAGAACTGAAGCGTGTCAACAAGC-3′; Igf2 shRNA-2: 5′-CCGGGTGGGCAAGT

TCTCCAATATCTCGAGATATTGGAAGAACTTGCCCACTTTTTG-3′, 5′-AATTCAA

AAAGTGGGCAAGTTCTTCCAATATCTCGAGATATTGGAAGAACTTGCCCAC-3′;

Igf2 shRNA-3: 5′-CCGGCTGATCGTGTTACCACCCAAACTCGAGTTTGGGTGGT

AACACGATCAGTTTTTG-3′, 5′-AATTCAAAAACTGATCGTGTTACCACCCAAACT

CGAGTTTGGGTGGTAACACGATCAG-3′; Igf2 shRNA-4: 5′-CCGGCAAAGA

GTTCAGAGAGGCCAACTCGAGTTGGCCTCTCTGAACTCTTTGTTTTTG-3′, 5′-AATTCAAAAACAAAGAGTTCAGAGAGGCCAACTCGAGTTGGCCTCTCTG

AACTCTTTG3′. The annealed oligonucleotides were ligated into the pLKO.1 vector and their sequences were verified. Then the four different Igf2 shRNAs were recombined into an adenoviral vector (AdEasy system) to generate recombinant adenovirus vector that expresses shRNA for Igf2, as previously described. Adenoviruses were generated by transfecting HEK 293A cells with vectors digested with *Pac*I according to the manufactuer’s instructions. Viruses were purified on a discontinuous CsCl gradient. Ad-shIGF2 expression plasmids were transfected into the hepa1-6 cell line to test the knockdown efficiency of these IGF2 shRNA constructs. The Ad-shIGF2 with the highest knockdown efficiency was selected for in vivo experiments. Mice received adenovirus via tail-vein injection at a dose of 1 × 10^9^ viral particles per gram body weight. A recombinant adenovirus vector that expresses a shRNA against GFP (Ad-shGFP) was used as a negative control.

### AAV8-IGF2 design and in vivo delivery

The IGF2 overexpression and control adeno-associated virus were generated by Obio Technology (Shanghai, China). The purified virus was diluted in sterile PBS. Viruses (5 × 10^11^) were injected through tail-vein at the volume of 100 µl 3 weeks before cell transplantation.

### Statistical analysis

All experiments were performed at least three times. Data were expressed as the mean ± standard deviation (s.d.). Statistical analyses were carried out using Graph Pad Prism 5.0c for Mac (Graph Pad Software). For parametric data, data significance was analyzed using a two-tailed unpaired Student’s *t*-test. In cases where more than two groups were being compared, then a one-way ANOVA was used. *F*-tests were used to compare variances between groups. *p* *<* 0.05 was considered statistically significant.

## Electronic supplementary material

Supplementary Files

## References

[1] Hill E, Boontheekul T, Mooney DJ (2006). Regulating activation of transplanted cells controls tissue regeneration. Proc. Natl Acad. Sci. USA.

[2] Soltys KA (2010). Barriers to the successful treatment of liver disease by hepatocyte transplantation. J. Hepatol..

[3] Huebert RC, Rakela J (2014). Cellular therapy for liver disease. Mayo Clin. Proc..

[4] Shafritz DA, Oertel M (2011). Model systems and experimental conditions that lead to effective repopulation of the liver by transplanted cells. Int. J. Biochem. Cell Biol..

[5] Oertel M, Menthena A, Dabeva MD, Shafritz DA (2006). Cell competition leads to a high level of normal liver reconstitution by transplanted fetal liver stem/progenitor cells. Gastroenterology.

[6] Menthena A (2011). p15INK4b signaling, and cell competition promotestem/progenitor cell repopulation of livers in aging rats. Gastroenterology.

[7] Serra MP (2012). Hepatocyte senescence in vivo following preconditioning for liver repopulation. Hepatology.

[8] Karnezis AN, Dorokhov M, Grompe M, Zhu L (2001). Loss ofp27 (Kip1) enhances the transplantation efficiency of hepatocytes transferred into diseased livers. J. Clin. Invest..

[9] Xiang D (2014). Non-viral FoxM1 gene delivery to hepatocytes enhances liver repopulation. Cell Death Dis..

[10] Overturf K (1996). Hepatocytes corrected by gene therapy are selected in vivo in a murine model of hereditary tyrosinaemia type I. Nat. Genet..

[11] Overturf K, al-Dhalimy M, Ou CN, Finegold M, Grompe M (1997). Serial transplantation reveals the stem-cell-like regenerative potential of adult mouse hepatocytes. Am. J. Pathol..

[12] Paulk NK (2012). In vivo selection of transplanted hepatocytes by pharmacological inhibition of fumarylacetoacetate hydrolase in wild-type mice. Mol. Ther..

[13] Wangensteen KJ, Zhang S, Greenbaum LE, Kaestner KH (2015). A genetic screen reveals Foxa3 and TNFR1 as key regulators of liver repopulation. Genes Dev..

[14] Vogel A (2004). Chronic liver disease in murine hereditary tyrosinemia type 1 induces resistance to cell death. Hepatology.

[15] Yoshiya S (2015). Blockade of the apelin-APJ system promotes mouse liver regeneration by activating Kupffer cells after partial hepatectomy. J. Gastroenterol..

[16] Padrissa-Altés S (2015). Control of hepatocyte proliferation and survival by Fgf receptors is essential for liver regeneration in mice. Gut.

[17] Kaldenbach M (2012). Hepatocyte growth factor/c-Met signaling is important for the selection of transplanted hepatocytes. Gut.

[18] Kimura M, Ogihara M (1998). Effects of insulin-like growth factor I and II on DNA synthesis and proliferation in primary cultures of adult rat hepatocytes. Eur. J. Pharmacol..

[19] Mogler C (2015). Hepatic stellate cell-expressed endosialinbalancesfibrogenesis and hepatocyte proliferation during liver damage. EMBO Mol. Med.

[20] Lamonerie T, Lavialle C, Haddada H, Brison O (1995). IGF-2 autocrine stimulation in tumorigenic clones of a human colon-carcinoma cell line. Int. J. Cancer.

[21] Foulstone E (2005). Insulin-like growth factor ligands, receptors, and binding proteins in cancer. J. Pathol..

[22] Krause P, Rave-Frank M, Christiansen H, Koenig S (2014). Preconditioning of the liver for efficient repopulation by primary hepatocyte transplants. Methods Mol. Biol..

[23] Kang LI, Mars WM, Michalopoulos GK (2012). Signals and cells involved in regulating liver regeneration. Cells.

[24] Stock P (2017). Impairment of host liver repopulation by transplanted hepatocytes in aged rats and the release by short-term growth hormone treatment. Am. J. Pathol..

[25] Lui JC, Finkielstain GP, Barnes KM, Baron J (2008). An imprinted gene network that controls mammalian somatic growth is down-regulated during postnatal growth deceleration in multiple organs. Am. J. Physiol. Regul. Integr. Comp. Physiol..

[26] Lui JC, Baron J (2013). Evidence that Igf2 down-regulation in postnatal tissues and upregulation in malignancies is driven by transcription factor E2f3. Proc. Natl Acad. Sci. USA.

[27] Chen DY (2011). A critical role for IGF-II in memory consolidation and enhancement. Nature.

[28] Pascual-Lucas M (2014). Insulin-like growth factor 2 reverses memory and synaptic deficits in APP transgenic mice. EMBO Mol. Med.

[29] Wang MJ (2014). Reversal of hepatocyte senescence after continuous in vivo cell proliferation. Hepatology.

[30] He ZY (2012). Murine embryonic stem cell-derived hepatocytes correct metabolic liver disease after serial liver repopulation. Int. J. Biochem. Cell Biol..

[31] Nagamoto Y (2016). Transplantation of a human iPSC-derived hepatocyte sheet increases survival in mice with acute liver failure. J. Hepatol..

[32] Turner RA (2013). Successful transplantation of human hepatic stem cells with restricted localization to liver using hyaluronan grafts. Hepatology.

