# Supplementary material for: Insulin-like growth factor 2 is a key mitogen driving liver repopulation in mice
Source: Cell Death Dis. 2018 Jan 18;9(2):26. doi: 10.1038/s41419-017-0186-1 (PMC5833551; doi:10.1038/s41419-017-0186-1)
Supplement: Supplementary file 1 — Supplementary Files [file 41419_2017_186_MOESM1_ESM.doc]

**Supplementary Materials and Methods**

**Immunohistochemistry (IHC) and Immunocytochemistry (ICC).** For immunohistochemistry staining, freshly harvested liver samples were fixed in 4% paraformaldehyde, embedded in paraffin, and sectioned into 2 μm-thick slices. The sections were de-paraffinized, rehydrated, and heated in a pressure cooker for 3 min at 121°C/100 kpa in citrate buffer (pH 6.0). The sections were incubated with primary antibodies at 4 °C overnight, then with secondary antibodies conjugated with fluorescent dye or HRP at 37 °C for 30 min. DAB (Vector Laboratories, Burlingame, CA) was applied on sections as substrate of the HRP for HRP-conjugated antibody. The sections were counterstained in hematoxylin (Sigma-Aldrich, St. Louis, MO) and covered in neutral balsam (Solarbio, Beijing, CHN). For immunocytochemical staining, cells were fixed with 4% paraformaldehyde for 10 min, permeabilized and blocked with blocking buffer (PBS containing 0.1% Triton X-100, 1% BSA) for 30 minutes. Cells were then incubated with primary antibody at 4 °C overnight, followed by fluorescence-conjugated secondary antibodies at 37 °C for 30 min. Nuclei were stained with 4’, 6-diamidino-2-phenylindole (DAPI) (Sigma-Aldrich, St. Louis, MO). All antibodies were listed in the Table 1. Images were acquired with a 50i Nikon fluorescence microscope (Nikon, Melville, NY). Images were processed with Adobe Photoshop CS4 software (San Jose, CA).

**Western Blot.** Total protein was extracted from cells using Total Protein Extraction Kit (Merck Millipore, Germany) according to manufacturer’s protocols. Proteins (50 µg) were separated on 10% or 12% SDS–polyacrylamide gels and electroblotted onto polyvinylidene fluoride (PVDF) membranes (Merck Millipore). Membranes were blocked with blocking buffer for 1 hour at room temperature and then incubated with primary antibodies at 4℃ overnight. Then, membranes were washed three times with PBST and incubated with HRP-conjugated secondary antibody at 37℃ for 30 min. Protein bands were detected by the SuperSignal West Pico Chemiluminescent Substrate (Thermo Fisher, NY). The list of primary and secondary antibodies is summarized as below.

**Primary antibodies used in the IHC and western blot assay**

| Primary Antibody | Brand | Cat. No. |
| --- | --- | --- |
| Tubulin | Abcam | Ab7291 |
| IGF-2 | Abcam | Ab9574 |
| Ki67 | eBioscience | 145698 |
| FAH | HepatoScience | HS602-910 hepatocytes |
| Cyclin D1 | Abcam | Ab134175 |
| Akt | CST | 4685 |
| p-Akt | CST | 4060 |
| ERK | CST | 4696 |
| p-ERK | CST | 5726 |
| BrdU | Abcam | ab6326 |
| Hnf4α | Santa Cruz | sc-8987 |
| IGF1R | CST | 3027 |
| P-IGF1R | CST | 6113 |

**RNA isolation, RT-PCR and quantitative PCR.** Total RNA from cells was isolated with Trizol reagent (Invitrogen, Carlsbad, CA) according to the manufacturer’s instructions. All reverse-transcriptase reactions were carried out with SuperScript II reverse transcriptase (Invitrogen) according to the manufacturer’s protocol. Specific primers were designed by Primer Premier 5. Quantitative PCR was performed in three repeats of each sample with ABI-7900 (Applied Biosystems Foster, City, CA) by SYBR Green master mix (Applied Biosystems). Fold change was calculated by the 2-ΔΔCT method. Detail informtion of Primers are listed below.

| Gene | Forward(5’→3’) | Reverse(5’→3’) | Tm  (℃) |
| --- | --- | --- | --- |
| IL6 | CTTCCATCCAGTTGCCTTCTT | CAAGTGCATCATCGTTGTTCAT | 55 |
| IL8 | CTCCTGCTGGCTGTCCTTAACC | GCCAACAGTAGCCTTCACCCAT | 55 |
| Tnfα | AACTGGCAGAAGAGGCACTCC | TGGGCTACAGGCTTGTCACTC | 58 |
| Tnfγ | GAACCAGCATCTTCCTCAG | CGGAGCGTGCAGTTCAGTGAT | 58 |
| Tgfβ | GACAAGGCTGATGTGGAAGG | GCTGCTCCATTCCGAACAC | 58 |
| Hgf | GCAGTCAGCACCATCAAGGCAA | TGGCACATCCACGACCAGGAA | 55 |
| Igf1 | CGTCTTCACACCTCTTCTACCT | GGCACAGTACATCTCCAGTCT | 58 |
| Igf2 | CAATATGACACCTGGAGACA | CTGATGGTTGCTGGACAT | 58 |
| Fgf | CGACTGCTGCTGGCTGTCTT | TTCTGGACTGCGGTGTGCTG | 60 |
| Egf | GGAACTGCGTCATGCTGGCTA | GTCCGCTGCTGCTCACACTT | 58 |

**ELISA.** ELISA experiments were all performed under manufacturer’s instructions (www.abcam.com).

**Supplementary Figure 1**


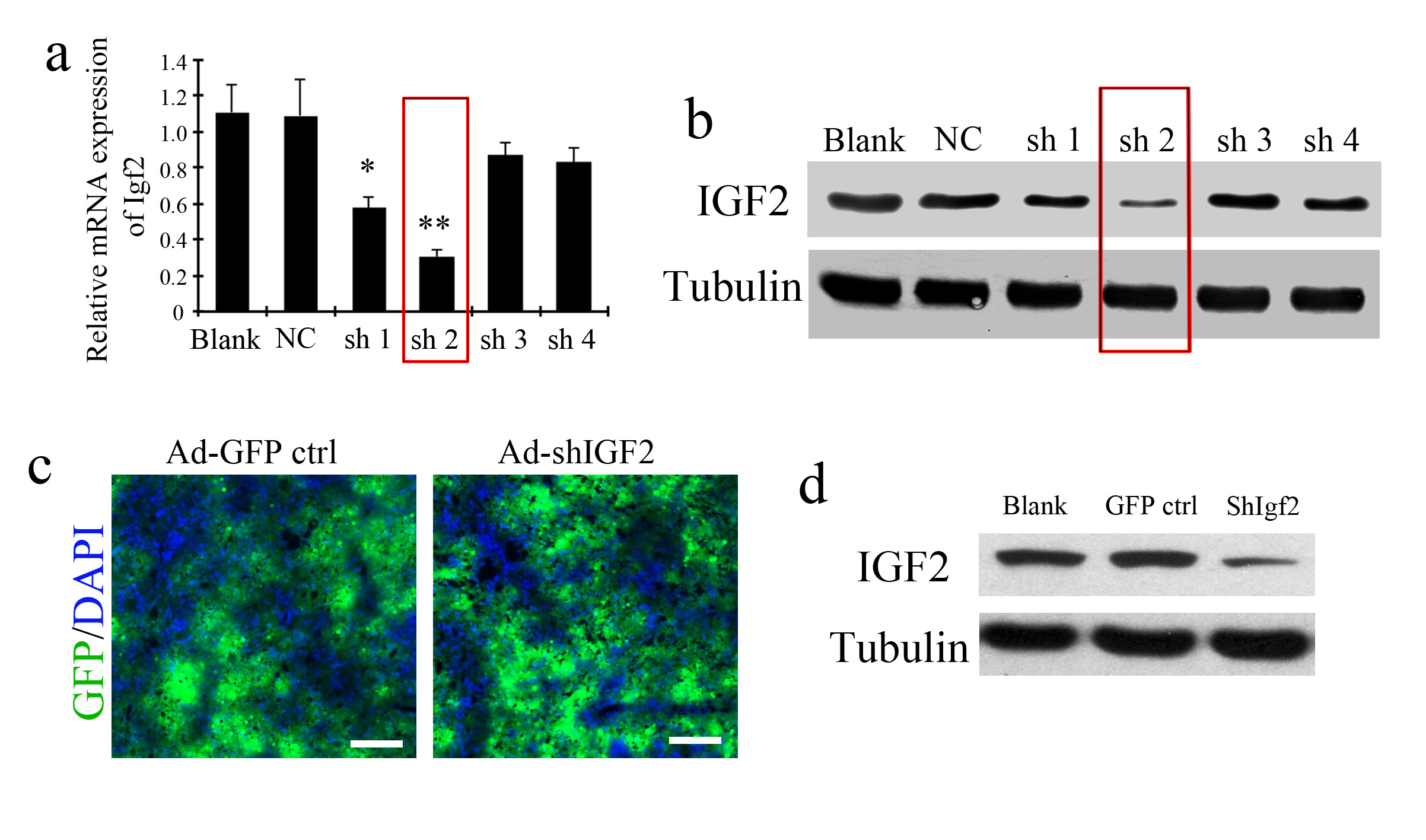


**The knockdown efficiency of Igf2 shRNA.**

(a) Quantitative mRNA expression of Igf2 in the cells without transfection and with transfection of adenoviruses-carried GFP control and four different sequences of ad-shIgf2. (b) Western blot analysis of IGF2 expression level in the cells without transfection and with transfection of adenoviruses-carried GFP control and four different sequences of ad-shIgf-2 adenoviruses. Red box indicated the selected Ad-shIgf2 with the higher knockdown efficiency. (c) The numbers of GFP-positive hepatocytes indicated the efficiency of adenoviruses infection in Fah^-/-^ mice withdrawal of NTBC one-week after infection. (d) The expression level of Igf2 in the livers of Fah^-/-^ mice withdrawal of NTBC after injection with Ad-GFP control or Ad-shIgf2 adenoviruses. Scale bar, 200 µm.

**Supplementary Figure 2**


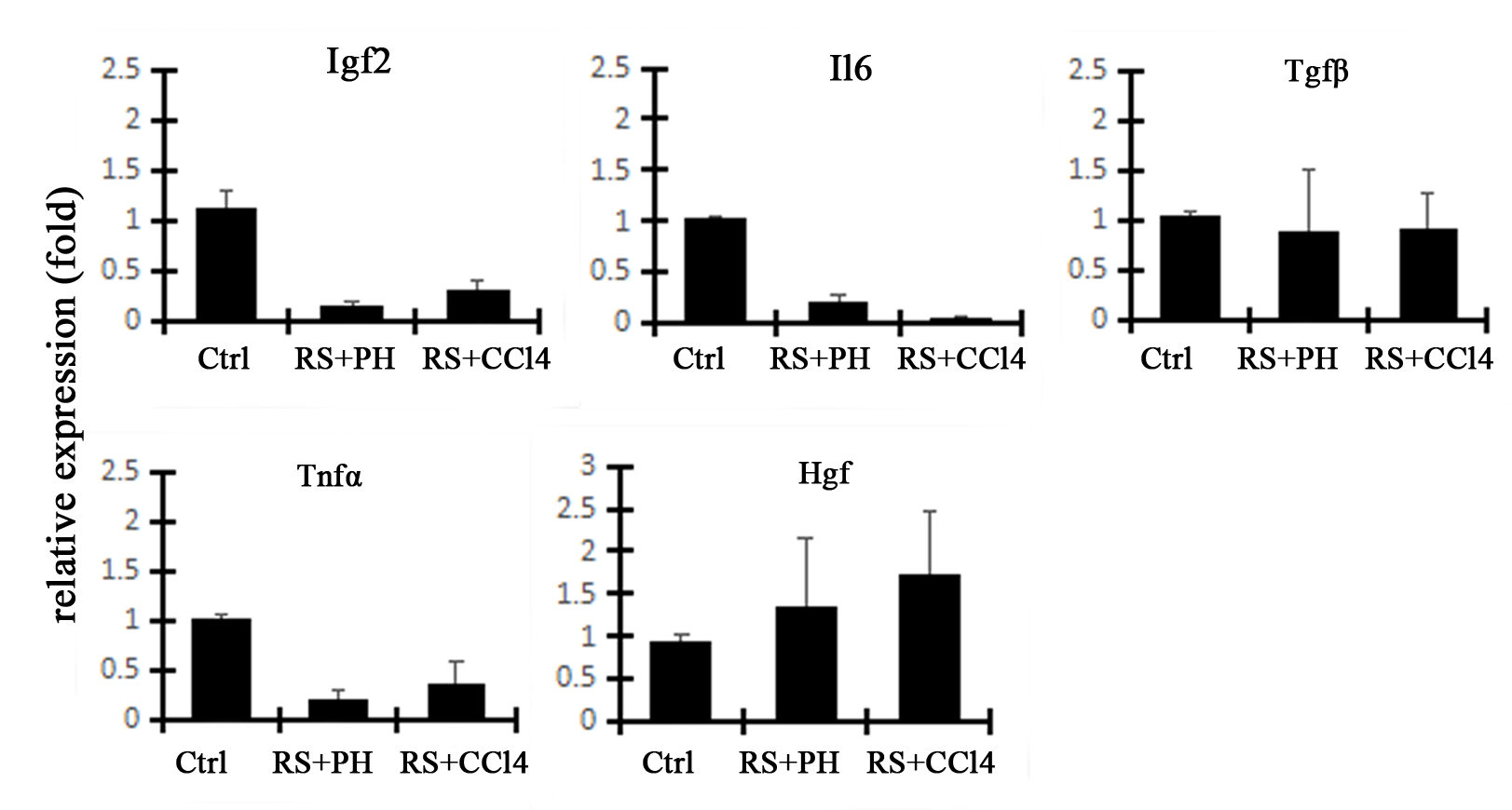


**Detection of cell factors and cytokines in RS-treated mice.**

Quantitation on the mRNA expressions of growth factors and cytokines in the livers of RS-treated mice.
